# Common immunological and prognostic features of lung and bladder cancer via smoking‐related genes: PRR11 gene as potential immunotherapeutic target

**DOI:** 10.1111/jcmm.18384

**Published:** 2024-05-18

**Authors:** YaXuan Wang, HaiXia Zhu, Lu Zhang, JiaXing He, Ji Bo, JianShe Wang, BeiChen Ding, MingHua Ren

**Affiliations:** ^1^ Department of Urology The First Affiliated Hospital of Harbin Medical University Harbin China; ^2^ Department of Central Laboratory Affiliated Tumor Hospital of Nantong University & Nantong Tumor Hospital Nantong China

**Keywords:** BLCA, immune checkpoint inhibitor, NSCLC, prognosis, PRR11

## Abstract

Smoking is a well‐known risk factor for non‐small‐cell lung cancer (NSCLC) and bladder urothelial carcinoma (BLCA). Despite this, there has been no investigation into a prognostic marker based on smoking‐related genes that could universally predict prognosis in these cancers and correlate with immune checkpoint therapy. This study aimed to identify smoking‐related differential genes in NSCLC and BLCA, analyse their roles in patient prognosis and immune checkpoint therapy through subgroup analyses, and shed light on PRR11 as a crucial prognostic gene in both cancers. By examining PRR11 co‐expressed genes, a prognostic model was constructed and its impact on immunotherapy for NSCLC and BLCA was evaluated. Molecular docking and tissue microarray analyses were conducted to explore the correlation between PRR11 and its reciprocal gene SPDL1. Additionally, miRNAs associated with PRR11 were analysed. The study confirmed a strong link between smoking‐related genes, prognosis, and immune checkpoint therapy in NSCLC and BLCA. PRR11 was identified as a key smoking‐associated gene that influences the efficacy of immune checkpoint therapy by modulating the stemness of these cancers. A prognostic model based on PRR11 co‐expressed genes in BLCA was established and its prognostic value was validated in NSCLC. Furthermore, it was found that PRR11 regulates PDL1 via SPDL1, impacting immunotherapeutic efficacy in both cancers. The involvement of hsa‐miR‐200b‐3p in the regulation of SPDL1 expression by PRR11 was also highlighted. Overall, the study elucidates that PRR11 modulates patient immunotherapy by influencing PDL1 expression through its interaction with SPDL1, with potential upstream regulation by hsa‐miR‐200b‐3p.

## 1 INTRODUCTION

Cancer is the second most prevalent reason behind global mortality. The biology of different cancer types is greatly impacted by exposure to risk factors.^1^ Understanding modifiable risk factors is crucial in managing cancer progression, and tobacco use and exposure to second‐hand smoke stand out as major contributors to this ailment. As outlined by the World Health Organization (WHO), approximately 1.1 billion people across the globe engage in tobacco consumption, resulting in an estimated 6 million deaths linked to tobacco every year.^2^ Cigarettes encompass deleterious constituents, including nicotine, acrolein, aromatic hydrocarbons, heavy metals and more than 7000 additional distinct chemicals. These substances play pivotal roles in the inflammatory and carcinogenic consequences of smoking.^3^

Tobacco smoke has been established to significantly impact the development of lung cancer by promoting the characteristics of cancer stem cells, while extracts from cigarette smoke have demonstrated the ability to induce epithelial–mesenchymal transition in human bladder cancer cells through activation of the ERK1/2 pathway.^4^, ^5^ The immune landscape within the tumour microenvironment (TME) has been widely recognized as a crucial determinant of prognosis and the antitumour immune response.^6^ Increasing evidence suggests that smoking cigarettes significantly affects the immune regulation. The remarkable anticancer impact of immune checkpoint blockade therapy has expanded the treatment repertoire for non‐small cell lung cancer. It has been observed that lung cancer patients who smoke exhibit a higher response rate to anti‐PD‐1 therapy compared with non‐smoking patients. Subsequent research has revealed that cigarette smoke can influence the response of NSCLC patients to immunotherapy by modulating oxidative phosphorylation and mitochondrial biogenesis.^7^ Regular smoking or exposure to second‐hand smoke weakens the effectiveness of immune cells in the immune system due to toxic substances. This can lead to the development of immunosuppressive components and the creation of an atypical immune microenvironment, ultimately promoting tumour growth.^8^ Lung cancer, frequently associated with smoking, is the most common type of cancer, accounting for 80%–90% of all smoking‐related cancer cases. Notably, casual smoking has been associated with a higher risk of developing health problems, including lung cancer, than abstaining from all smoking practices.^9^ Similarly, smoking continues to be the main risk factor for bladder cancer and is associated with unfavourable clinical and cancer‐related outcomes.^10^ Accurate prediction of prognosis and therapeutic response is crucial for optimizing treatment strategies and improving patient outcomes.^11^ However, to date, no research has examined the existence of a smoking‐associated gene‐based prognostic marker that could aid in the prognosis of both bladder and lung cancers, as well as being linked to immunotherapy for these cancers.

The rapid development of bioinformatics in recent years has significantly improved the diagnosis and prognosis of diseases.^12^ This study aimed to use bioinformatics methods to analyse the presence of common immune‐related prognostic genes in lung and bladder cancer, with a particular emphasis on smoking‐related genes. We classified samples from the cancer genome atlas (TCGA) bladder urothelial carcinoma (BLCA) and non‐small cell lung cancer (NSCLC) datasets into two groups based on smoking habits. Our analysis revealed a significant correlation between smoking‐associated genes, immune infiltration, and immune checkpoints in both NSCLC and BLCA. This suggests that these smoking‐associated genes may play a role in the regulation of immunotherapy based on subgroup typing and least absolute shrinkage and selection operator (LASSO). Furthermore, we identified PRR11 as a common immune‐related prognostic gene in patients with NSCLC and BLCA. PRR11 regulates PDL1 expression by interacting with SPDL1 in both types of cancer. Additionally, our findings revealed a negative correlation between hsa‐miR‐200b‐3p and the expression of PRR11, SPDL1, and PDL1 in NSCLC and BLCA. This suggests that hsa‐miR‐200b‐3p is involved in the regulation of PDL1 by PRR11 via SPDL1. In conclusion, our study highlights the importance of the hsa‐miR‐200b‐3p/PRR11/SPDL1 signalling axis in the context of immunotherapy for NSCLC and BLCA, specifically in the regulation of PDL1.

## 2 MATERIALS AND METHODS

### 2.1 Samples, datasets and antibody

Clinical information on the 406 BLCA and 1017 NSCLC samples and RNA‐seq data were obtained from the TCGA database (https://portal.gdc.com). Bladder and lung cancer tissue microarrays were acquired from Shanghai Outdo Biotech Company. Sixty lung cancer tissue microarrays and 41 bladder samples were included in this study. This study was approved by the Shanghai Outdo Ethics Committee. PRR11 (bs‐6237R) and SPDL1 (bs‐2321R) antibodies were purchased from BIOSS.

### 2.2 Analysis of differences in smoking‐related genes

NSCLC and BLCA samples were classified into smoking and non‐smoking groups. For bladder cancer, there were 286 and 109 samples from the smoking and non‐smoking groups, respectively. For lung cancer, there were 896 and 92 samples from the smoking and non‐smoking groups, respectively. Differential expression of mRNAs was investigated using the Limma package in R software (version 3.40.2.). We defined ‘*p* < 0.05, and log2 (fold change) > 1.3 or log2 (fold change) < −1.3’ as the threshold mRNA differential expression screening.^13^

### 2.3 Consistency cluster analysis

Consistency analysis was conducted on the NSCLC and BLCA clinical and RNA‐seq data obtained from TCGA. The ConsensusClusterPlus R package version 1.54.0 was employed for this purpose. To perform the analysis, a maximum of six clusters were defined. Additionally, 80% of the total sample was randomly selected 100 times, using clusterAlg = ‘hc’ and innerLinkage = ‘ward.D2’. To visualize the clustering results, we utilized the pheatmap R software package version 1.0.12 to generate cluster heat maps. All statistical analyses were carried out using R version 4.0.3. For analysing the survival patterns, Kaplan–Meier (KM) survival curves were generated for different subgroups of samples within the dataset, and log‐rank tests were used to compare the survival outcomes between groups.

### 2.4 Analysis of immune infiltration and Immune Checkpoint Blockade therapy responsiveness

To reliably evaluate the immune score, we utilized immuneeconv, an R software package that assimilates six contemporary algorithms: TIMER, xCell, MCP‐counter, CIBERSORT, EPIC and quanTIseq. The six algorithms were benchmarked, and each exhibited a distinctive edge.^14^ In this study, we chose the CIBERSORT algorithm because it can analyse a relatively large number of immune‐infiltrating cells.^15^ The tumour immune dysfunction and exclusion (TIDE) algorithm evaluates two distinct mechanisms of tumour immune escape: CTL dysfunction in tumour‐infiltrating cells and CTL rejection by immune‐suppressing factors. Tumours with high TIDE scores exhibit poor responsiveness and lower survival rates following Immune Checkpoint Blockade therapy. This algorithm uses a set of gene expression markers to assess these mechanisms.^16^ The Tumour Immunization Single Cell Center (TISCH) database was used to analyse the correlation between PRR11 and immune infiltration in NSCLC and BLCA.^17^

### 2.5 Gene function analysis

In order to authenticate the prospective roles of these targets, we undertook an analysis dedicated to functional enrichment. We utilized the Gene Ontology (GO) database to annotate genes according to their molecular functions (MF), biological pathways (BP) and cellular components (CC). Moreover, we performed an analysis on gene functions and relevant high‐level genomic functional information using the Kyoto Encyclopedia of Genes and Genomes (KEGG) enrichment analysis.^18^ To acquire a more profound comprehension of the oncogenic functions of the target genes, we implemented the ClusterProfiler package in R to assess the GO functionality of potential mRNAs and to enrich the KEGG pathways. Additionally, the ClusterProfiler tool offers the capability to execute gene set enrichment analysis (GSEA).^19^, ^20^

### 2.6 Correlation analysis of PRR11 with stemness characteristics of NSCLC and BLCA

The mRNA signature was determined using the one‐class linear regression (OCLR) algorithm established by Malta et al. A compilation of 11,774 genes formed the gene expression profile, which was established according to mRNA expression patterns. To transform RNA expression data into a comparable scale, Spearman's correlation analysis was performed using the aforementioned technique. Subsequently, the dryness index was assigned to the interval [0,1] by subtracting the smallest value and dividing by the largest value.^21^, ^22^ All analytical techniques and R packages employed in this study were performed using R Foundation for Statistical Computing (2020) version 4.0.3.

### 2.7 Construction of a prognostic model based on PRR11 co‐expressed genes

Initially, we detected predictive genes displaying correlation coefficients exceeding 0.5 for PRR11. Following that, we applied the LASSO regression algorithm to select features, conducted 10‐fold cross‐validation and employed the R package glmnet to conduct the analysis. A predictive model was formulated by utilizing the LASSO algorithm. KM survival analysis was executed using the log‐rank test to compare differences in survival between the aforementioned groups. These analyses and the R package were conducted employing R software version 4.0.3 (R Foundation for Statistical Computing, 2020).

### 2.8 Molecule docking

Protein–protein docking was performed to examine the relationship between PRR11 and SPDL1. We used the Protein Data Bank (PDB) (http://www.rcsb.org/) to obtain the PDB format for the protein domain. Subsequently, the ZDOCK module assigned docking sites and computed the corresponding ZDOCK scores. Autodock Vina 1.2.2 (http://autodock.scripps.edu/) was employed for the molecular docking studies.

### 2.9 Immunohistochemical analysis of PRR11 and SPDL1 expression in NSCLC and BLCA tissues

After removing the wax from the microarrays, a peroxidase inhibitor was applied to the tissue and incubated at room temperature for 10 min to repair the antigen. Once the sealing process was completed, the PRR11 antibody (with a dilution ratio of 1:40) was introduced and left to incubate overnight at a temperature of 4°C. Subsequently, the PRR11 antibody was washed off and the tissue underwent a 30‐min incubation with the secondary antibody. Finally, colour development, restoration and sealing were performed. The SPDL1 dilution ratio was 1:40. The staining results were independently evaluated by two pathologists who employed numerical scales of 1, 2 and 3 to indicate low, medium and high concentrations, respectively. The staining patterns were further categorized into the following ranges: 0%–25%, 26%–50%, 51%–75% and 76%–100%, which were correspondingly assigned scores of 1, 2, 3 and 4. Finally, the total score was obtained by multiplying the scores of the two evaluations.

### 2.10 Statistical analysis

The Wilcoxon test was used to assess statistical differences between two groups. The Kaplan–Meier curves underwent a log‐rank test for survival analysis, and any *p*‐value < 0.05 was considered statistically significant.

## 3 RESULTS

### 3.1 Identification of smoking‐related genes in BLCA and NSCLC

**FIGURE 1:**
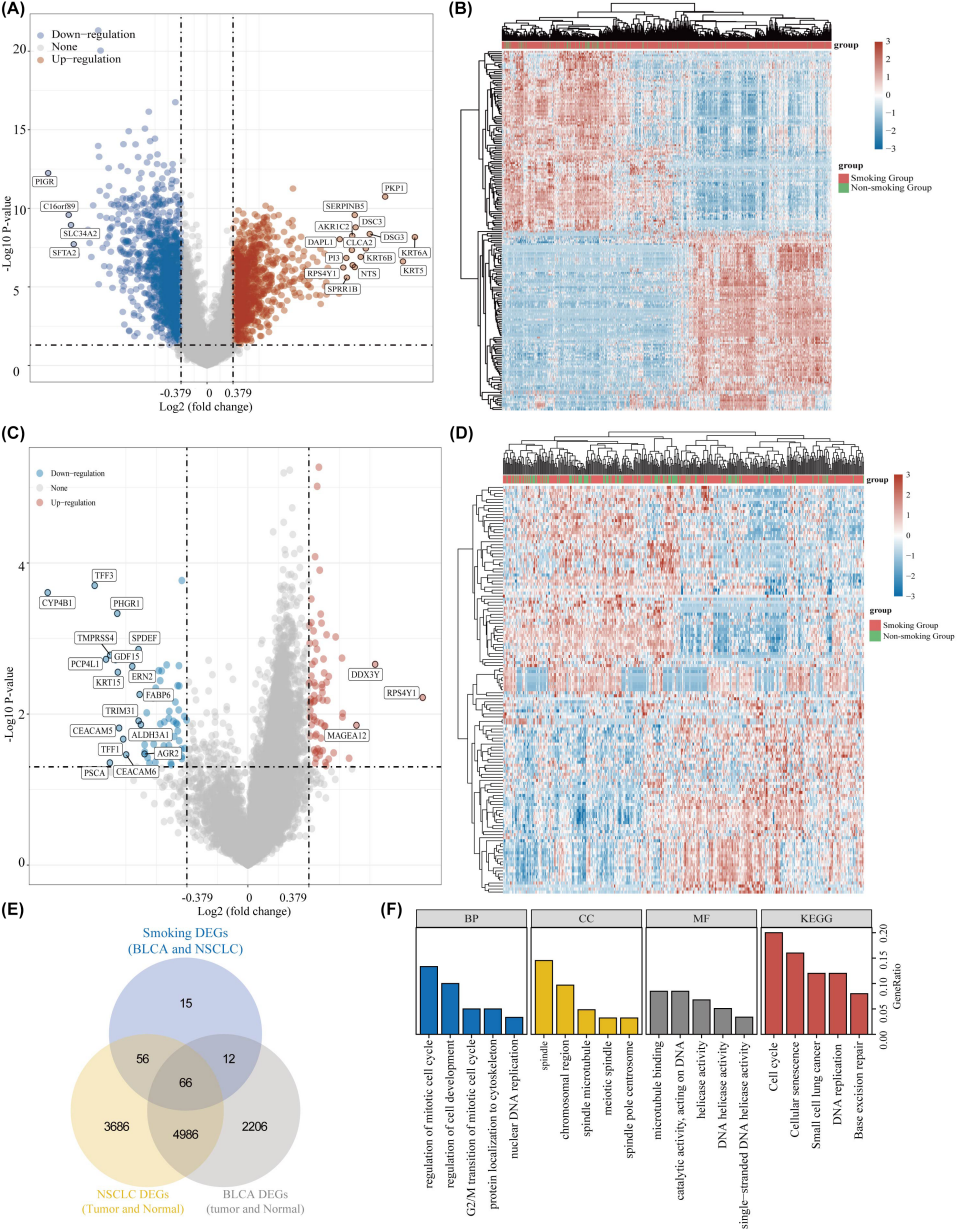
Sixty‐six genes identified as smoking‐related genes in BLCA and NSCLC. (A, B) Differential analyses based on smoking and non‐smoking samples in NSCLC. (C, D) Differential analyses based on smoking and non‐smoking samples in BLCA. (E) Venn diagram of common smoking‐related differential genes in BLCA and NSCLC. (F) Gene enrichment analysis of smoking‐related differential genes.

Smoking habit has been extensively investigated to elucidate the molecular mechanisms underlying its potential to promote BLCA and NSCLC. For this purpose, we thoroughly examined BLCA and NSCLC samples obtained from the TCGA database (Figure 1A–D). The enrolled patients were categorized into ‘smokers’ and ‘non‐smokers’. Through comparative analysis of gene expression in BLCA and NSCLC tissues with their respective normal tissues from the TCGA database, we successfully identified 66 differentially expressed genes that were associated with smoking (Figure 1E). Thereafter, enrichment analysis of these 66 genes was conducted to elucidate their probable biological functions. KEGG analysis indicated a strong correlation between these genes and crucial cellular processes, such as the cell cycle, cellular senescence, DNA replication and base excision repair. Moreover, GO analysis yielded significant evidence supporting the association of these genes with cell‐cycle regulation and DNA repair (Figure 1F).

### 3.2 Cluster analysis of NSCLC and BLCA samples

**FIGURE 2:**
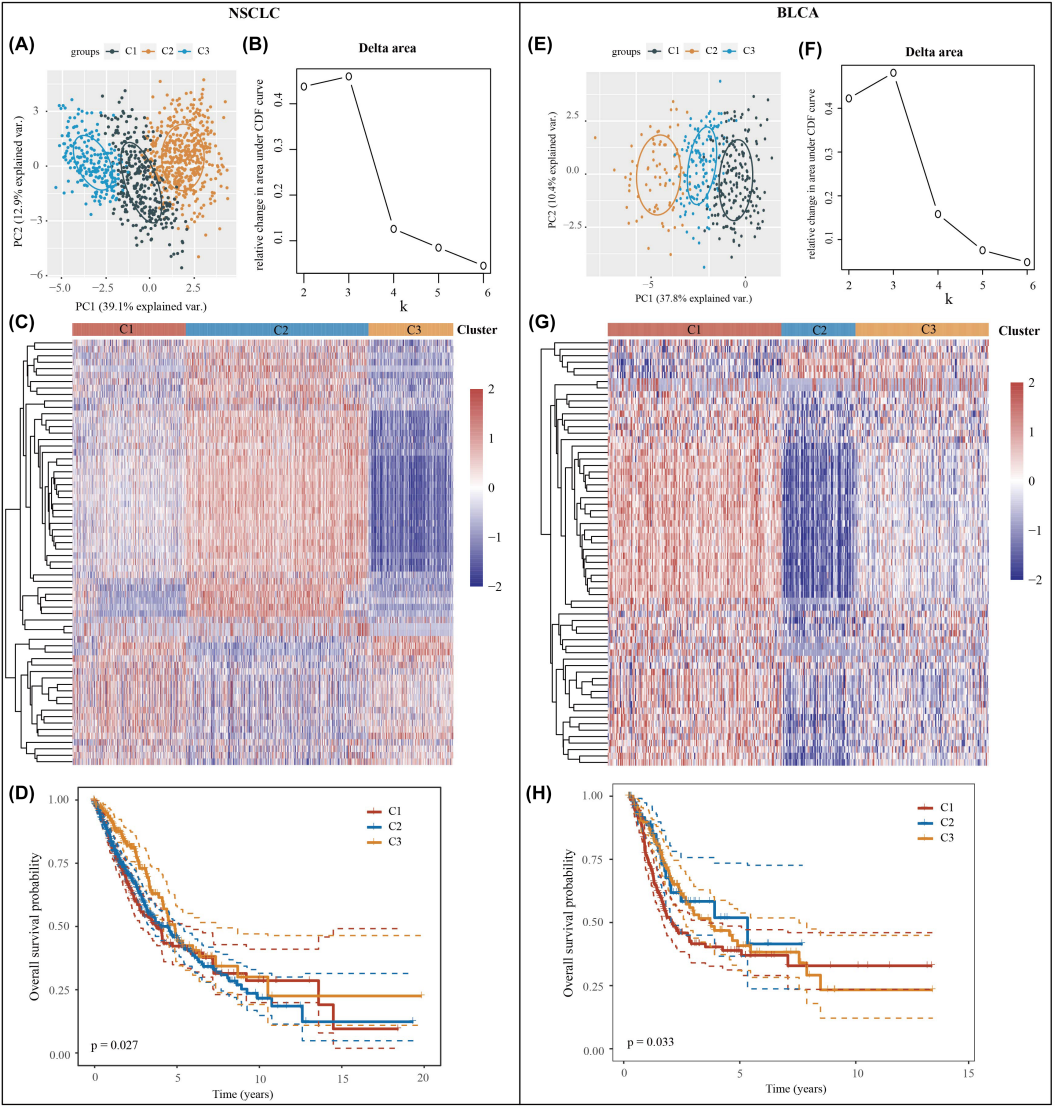
Subgroup typing for NSCLC and BLCA samples. (A) Sample distribution of the three clusters in NSCLC. (B) CDF Delta area. (C) Heatmap of the expression of smoking‐related genes in three NSCLC clusters. (D) Overall survival for three NSCLC clusters. (E) Sample distribution of the three clusters in BLCA. (F) CDF Delta area. (G) Heatmap of the expression of smoking‐related genes in three BLCA clusters. (H) Overall survival for three BLCA clusters.

To further investigate the impact of smoking‐related genes on the prognosis and immune infiltration in NSCLC and BLCA, we conducted subgroup analyses of NSCLC and BLCA samples. We divided the NSCLC samples into three clusters and their distribution maps can be observed in Figure 2A,B. The heat map provides a detailed comparison of the expression of 66 genes across the three clusters. Notably, more than half of the genes exhibited higher expression in Cluster 2 and lower expression in Cluster 3 (Figure 2C). Additionally, we assessed the overall survival of the three clusters. The results revealed significant differences in overall survival between the clusters (Figure 2D). Moreover, we examined the correlation between the three clusters and clinicopathological factors of NSCLC, as detailed in Table 1. We also conducted cluster analyses of BLCA samples to investigate the impact of smoking‐related genes on the prognosis and immune infiltration in patients with BLCA. To maintain consistency with the NSCLC analyses, we classified the BLCA samples into three clusters based on the inflection point in the delta area at *k* = 3 (Figure 2E,F). By examining the expression of smoking‐related genes using a heat map, we observed that more than half of the genes were highly expressed in Cluster 1 and less expressed in Cluster 2 (Figure 2G). Furthermore, we analysed overall survival in the three bladder cancer clusters and found the same significant differences in overall survival prognosis between the three clusters (Figure 2H). Additionally, we explored the correlation between these three clusters and clinicopathological factors in patients with BLCA (Table 2). These findings highlighted the significance of smoking‐related genes in predicting the prognosis of patients with NSCLC and BLCA.

**TABLE 1:** Correlation analysis of three subtypes with sex, age and stage in NSCLC samples.

	Feature	C1	C2	C3	*p*‐Value
Status	Alive	176	280	158	
	Dead	127	208	68	0.004
Gender	Female	141	125	142	
	Male	162	363	84	0
pT_stage	T1	40	44	33	
	T1a	19	23	29	
	T1b	27	36	32	
	T2	107	175	59	
	T2a	49	88	32	
	T2b	17	36	8	
	T3	30	65	23	
	T4	12	21	9	
	TX	2		1	0
pN_stage	N0	186	304	161	
	N1	67	125	35	
	N2	43	50	21	
	N3	2	5		
	NX	5	4	8	0.003
pM_stage	M0	210	390	158	
	M1	11	9	3	
	M1a	2	1		
	M1b	2	3	1	
	MX	77	81	61	0.006
pTNM_stage	I	4	3	1	
	IA	71	74	76	
	IB	77	151	63	
	II	1	3		
	IIA	32	64	19	
	IIB	51	85	29	
	III	1	2		
	IIIA	42	71	23	
	IIIB	8	17	4	
	IV	15	13	5	0

**TABLE 2:** Correlation analysis of three subtypes with sex, age and stage in BLCA samples.

	Feature	C1	C2	C3	P‐value
Status	Alive	90	57	81	
	Dead	95	22	61	0.002
Gender	Female	50	18	38	
	Male	135	61	104	0.754
pT_stage	T1	4	2	5	
	T2	46	30	34	
	T2a	14	6	11	
	T2b	26	6	17	
	T3	19	13	14	
	T3a	22	5	19	
	T3b	33	5	27	
	T4	4	1	3	
	T4a	13	8	11	
	T4b	2			
	TX	2	3	1	0.246
pN_stage	N0	110	53	73	
	N1	23	5	18	
	N2	27	14	34	
	N3	5		2	
	NX	17	7	12	0.228
pM_stage	M0	73	52	70	
	M1	7	1	3	
	MX	103	26	68	0.004
pTNM_stage	II	51	36	42	
	III	74	22	44	
	IV	59	20	54	
	I		1	1	0.02

### 3.3 Smoking‐related genes correlate with the immune microenvironment in both NSCLC and BLCA

**FIGURE 3:**
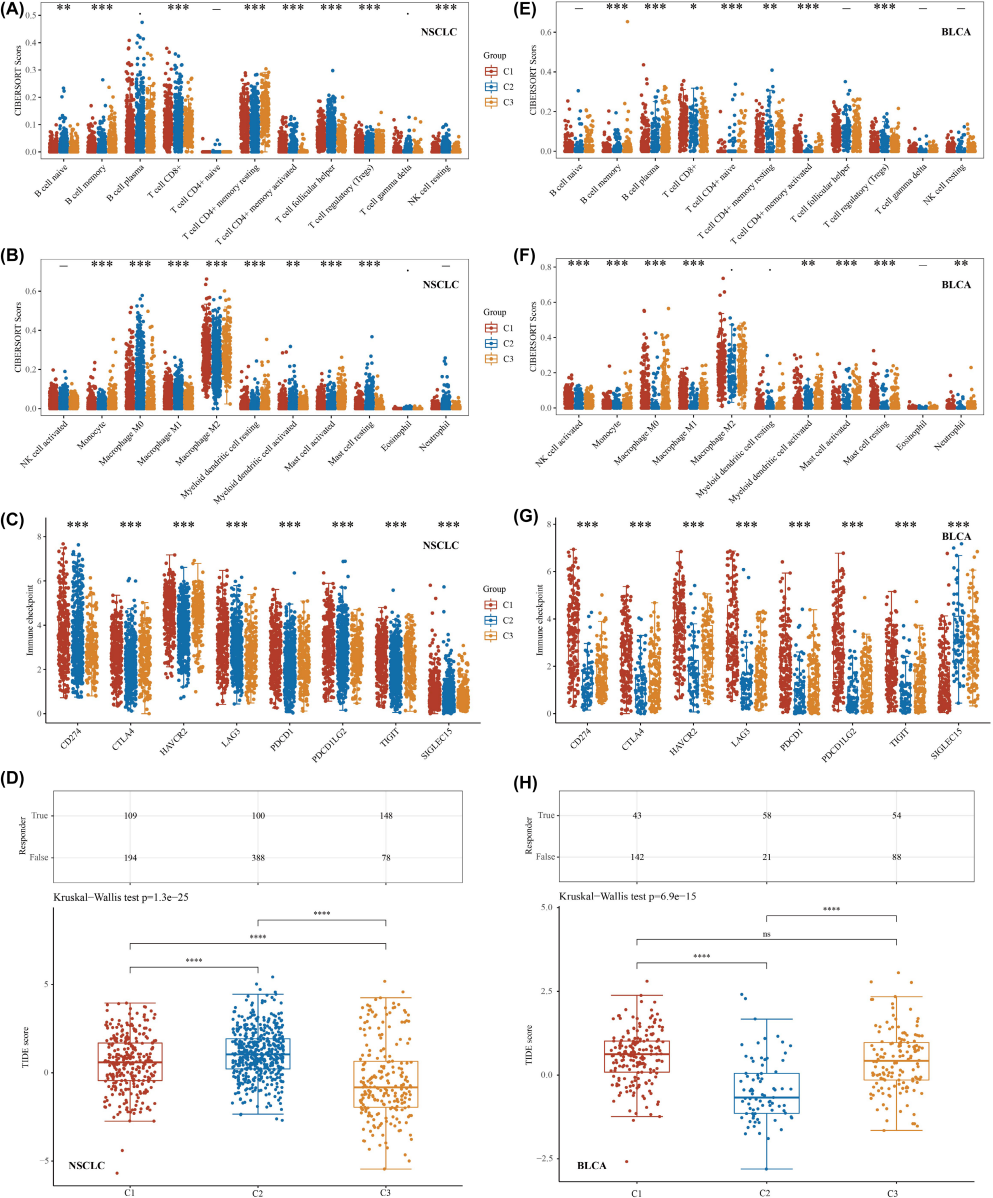
Smoking‐related genes significantly associated with immunotherapy for NSCLC and BLCA. (A, B) Differential expression of immune‐infiltrating cells in lung cancer in the three clusters analysed based on the CIBERSORT algorithm. (C) Differential expression of immune checkpoint‐related genes in the three clusters in NSCLC. (D) Predicting immune checkpoint inhibitor treatment response using the TIDE algorithm in NSCLC. (E, F) Differential expression of immune‐infiltrating cells in bladder cancer in three clusters analysed based on the CIBERSORT algorithm. (G) Differential expression of immune checkpoint‐related genes in the three clusters in BLCA. (H) Predicting immune checkpoint inhibitor treatment response using the TIDE algorithm in BLCA. *p < 0.05, **p < 0.01 and ***p < 0.001.

Immunotherapies have arisen as a highly promising and crucial approach to treating cancer, greatly influencing the treatment outcomes of many types of solid tumours.^23^ In this study, we used the CIBERSORT algorithm to analyse the correlation between the three clusters based on the composition of smoking‐related genes and immune infiltration in lung cancer. Our findings revealed that the levels of B cell plasma, naive CD4+ T cells, gamma delta T cells, activated NK cells, eosinophils and neutrophils were not significantly different among the three clusters. However, the levels of the remaining 16 immune infiltration‐associated cells varied significantly among clusters (Figure 3A,B). Additionally, we examined the expression of immune checkpoint‐related genes in the three clusters. Our results demonstrated that all immune checkpoint‐related genes displayed significant differences among the clusters, with Cluster 1 consistently exhibiting the highest expression levels (Figure 3C). The TIDE algorithm was used to predict the responsiveness of the three clusters to immune checkpoint inhibitor therapy. Cluster 3 exhibited the lowest TIDE scores, indicating that patients in Cluster 3 had the most favourable response to immune checkpoint inhibitor treatment. In contrast, Clusters 1 and 2 demonstrated poorer responses to immune checkpoint inhibitor treatment than Cluster 3 did (Figure 3D). In bladder cancer samples, B‐cell naive, T‐cell follicular helper, T‐cell gamma delta, NK‐cell resting, macrophage M2, myeloid dendritic cell resting and eosinophil scores did not significantly differ among the three clusters. However, the remaining 15 immune infiltration‐associated cells exhibited significantly different scores among the three clusters (Figure 3E,F). The expression of immune checkpoint‐related genes also varied significantly across all three BLCA clusters, with SIGLEC15 highly expressed in Cluster 2, and all other immune checkpoint‐related genes highly expressed in Cluster 1 (Figure 3G). Finally, we evaluated the responsiveness of the three clusters to immune checkpoint inhibitor treatment and found that patients in Cluster 2 had the lowest TIDE scores, indicating that Cluster 2 had the most favourable outcomes after receiving immune checkpoint inhibitor treatment (Figure 3H). Collectively, these findings support the strong association between smoking‐related genes and immunotherapy in NSCLC and BLCA.

### 3.4 PRR11 in smoking‐related genes as a prognostic gene associated with immune infiltration in both NSCLC and BLCA

**FIGURE 4:**
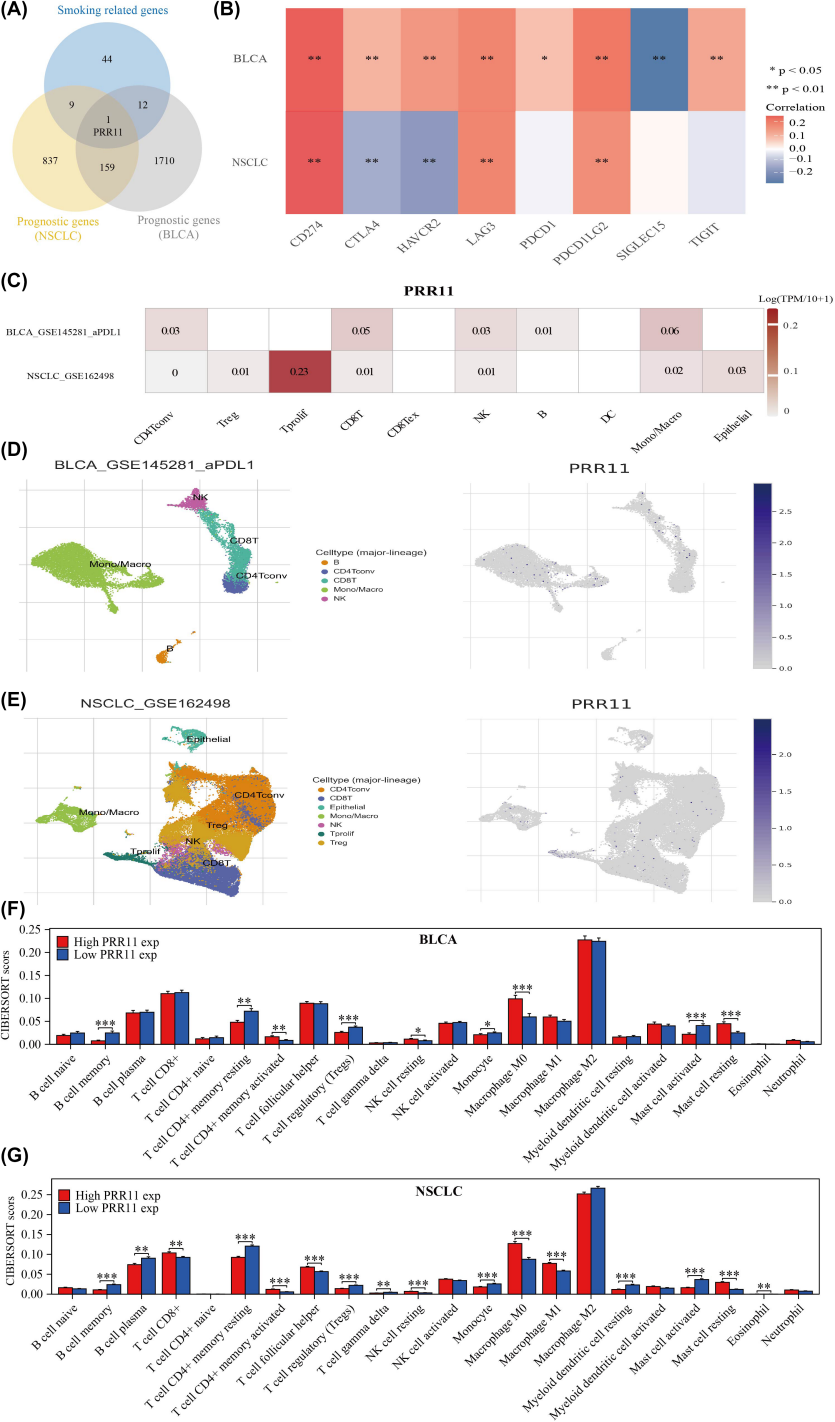
PRR11 is strongly associated with immunotherapy for lung and bladder cancer through regulation of monocyte and macrophage M0. (A) Venn diagram mapping the intersection of smoking‐related genes with NSCLC and BLCA prognostic genes. (B) PRR11 correlates with immune checkpoints in NSCLC and BLCA. (C–E) Single‐cell analysis of PRR11 correlates with immune infiltration correlation. (F, G) Correlation of PRR11 with immune infiltration in NSCLC and BLCA in the TCGA dataset. *p < 0.05, **p < 0.01 and ***p < 0.001.

To further screen for the best immune‐related prognostic genes in NSCLC and BLCA, we combined the NSCLC and BLCA prognostic genes. Our analysis evidenced that PRR11 may be a common prognostic gene in both NSCLC and BLCA (Figure 4A). In BLCA, PRR was positively correlated with CD274, CTLA4, HAVCR2, LAG3, PDCD1, PPDCD1LG2 and TIGIT, and negatively correlated with SIGLEC15. In NSCLC, PRR was found to have a positive correlation with CD274, LAG3 and PPDCD1LG2, a negative correlation with CTLA4 and HAVCR2, and no significant correlation with PDCD1, SIGLEC15 and TIGIT. Interestingly, in both NSCLC and BLCA, PRR was positively correlated with CD274, LAG3 and PDCD1LG2, with CD274 exhibiting the strongest correlation (Figure 4B). Subsequently, we analysed the relevance of PRR in immune infiltration in NSCLC and BLCA using single‐cell analysis. The results revealed that PRR correlated with CD8T, NK, and Mono/Macro in both NSCLC and BLCA (Figure 4C). By examining the cluster plots of single‐cell RNA sequencing data, we determined the location and expression of PRR11 in various immune cells (Figure 4D,E). Finally, we used the CIBERSORT algorithm to analyse the correlation between PRR11 and immune infiltration in NSCLC and BLCA using TCGA dataset. Our findings revealed that PRR11 was significantly associated with memory B cells, T‐cell CD4+ memory resting, T‐cell CD4+ memory activated, regulatory T cells (Treg), resting NK cells, monocytes, M0 macrophages, activated mast cells and resting mast cells in both NSCLC and BLCA (Figure 4F,G). In conclusion, our study confirmed the significant correlation between PRR11 expression and immune cell infiltration in both NSCLC and BLCA. Based on the conclusions drawn from the single‐cell analysis and CIBERSORT algorithm, we suggest that PRR11 may play a role in the immune microenvironment of NSCLC and BLCA through the regulation of monocytes and M0 macrophages.

### 3.5 PRR11 is associated with stemness characteristics in BLCA and NSCLC

**FIGURE 5:**
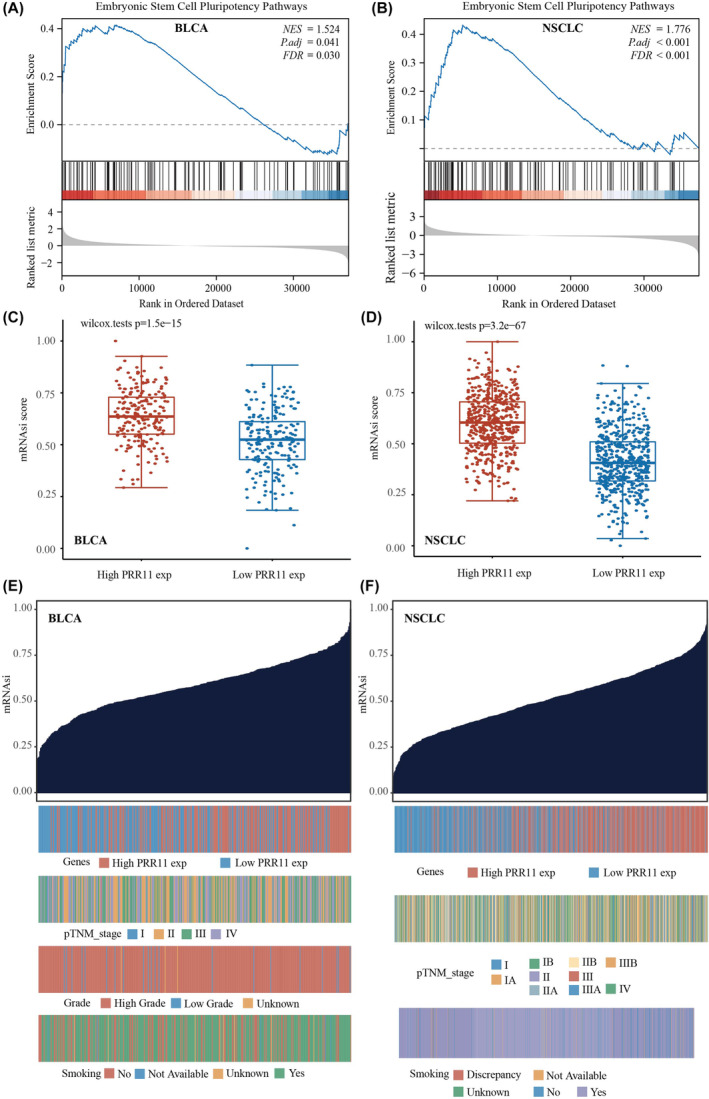
PRR11 positively correlates with stemness score in BLCA and NSCLC. (A) PRR11 is significantly associated with embryonic stem cell pluripotency pathways in BLCA. (B) PRR11 is significantly associated with embryonic stem cell pluripotency pathways in NSCLC. (C) PRR11 positively correlates with stemness score in BLCA. (D) PRR11 positively correlates with stemness score in NSCLC. (E) PRR11 expression, clinical stage, grading, smoking history and stemness score distribution in BLCA. (F) PRR11 expression, clinical stage, smoking history and dryness score distribution in NSCLC.

Stemness plays a crucial role in enabling tumour cells to resist various treatments. Using GSEA (Figure 5A,B), we discovered a significant association between PRR11 expression and stemness in both BLCA and NSCLC. To further investigate this, we examined the correlation between PRR11 and stem cell characteristics in BLCA and NSCLC. Our findings revealed that stemness scores were consistently higher in the PRR11 high‐expression group than in the PRR11 low‐expression group in both BLCA and NSCLC. This suggests that elevated PRR11 expression is correlated with increased stemness characteristics in BLCA and NSCLC and that it promotes the stemness characteristics of these cancers (Figure 5C,D). Furthermore, we analysed the distribution of stemness scores in BLCA and NSCLC samples, considering the clinical stage, grading and smoking history of the patients (Figure 5E,F).

### 3.6 Construction of a prognostic model based on PRR11 co‐expressed genes

**FIGURE 6:**
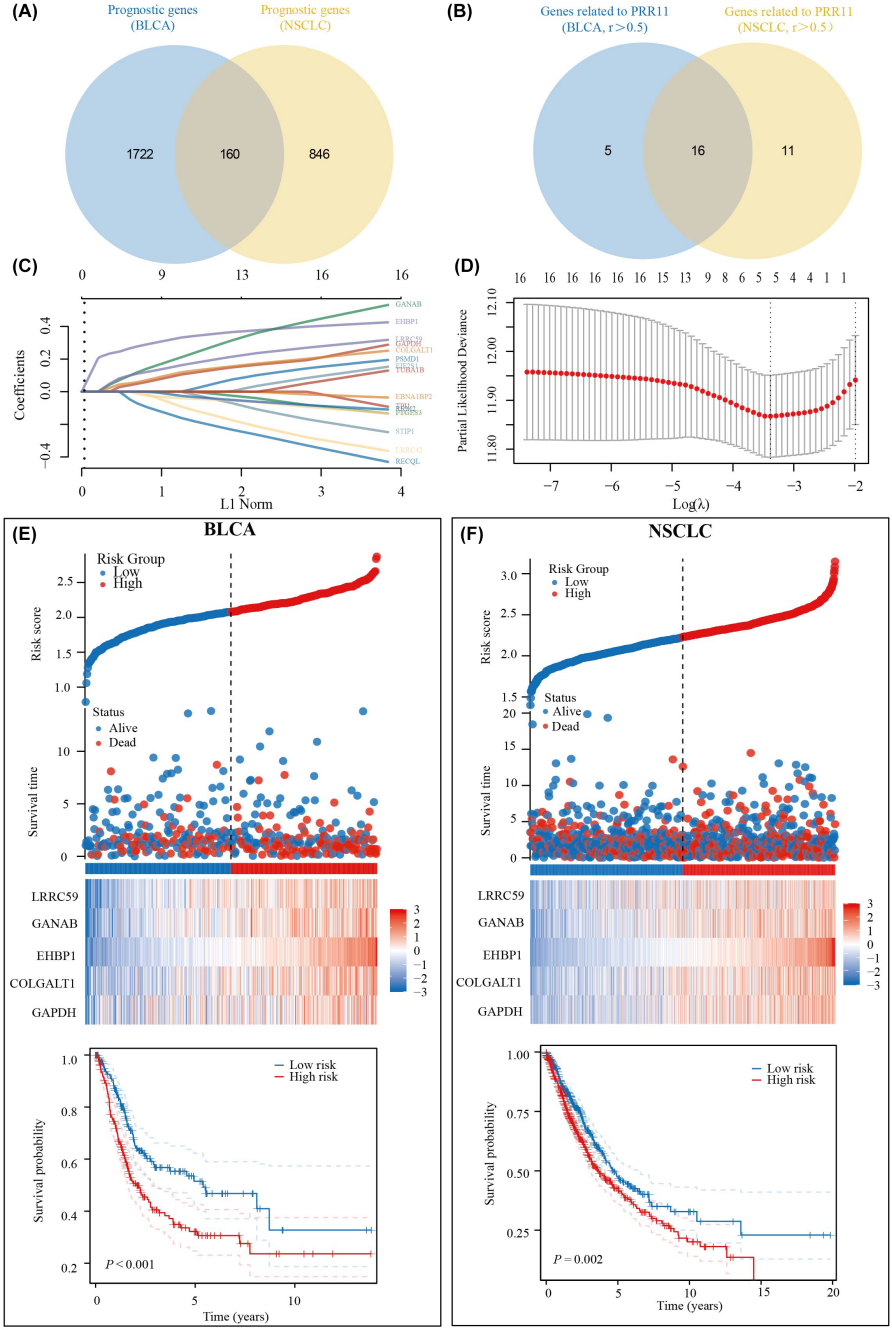
Prognostic model simultaneously predicts prognosis of BLCA and NSCLC patients based on co‐expressed PRR11 gene. (A) Common prognostic genes in BLCA and NSCLC. (B) Genes associated with PRR11 in BLCA and NSCLC. (C, D) Five genes co‐expressed with PRR11 were included in the prognostic model. (E) Risk score and survival time, survival status and prognostic differences between high‐ and low‐risk groups in BLCA. (F) Risk score and survival time, survival status and prognostic differences between high‐ and low‐risk groups in NSCLC.

Initially, we screened for prognostic genes common to BLCA and NSCLC (Figure 6A). Of the 160 genes identified, 16 exhibited correlation coefficients >0.5 with PRR11 in both BLCA and NSCLC (Figure 6B). Subsequently, these 16 genes were included in the analysis using the LASSO algorithm in BLCA, resulting in the identification of five genes for inclusion in the model (Figure 6C,D). The risk score was calculated based on the expression levels of GAPDH, COLGALT1, EHBP1, GANAB and LRRC59 in BLCA. Patients in the high‐risk group had a significantly worse prognosis than those in the low‐risk group (Figure 6E). Subsequently, this model was applied to lung cancer samples to investigate whether it could predict the prognosis of patients with lung cancer. The results confirmed that the model could predict the prognosis of patients with lung cancer, with those in the high‐risk group having a significantly worse prognosis than those in the low‐risk group (Figure 6F). Therefore, we concluded that the prognostic model constructed using the PRR11 co‐expression gene could simultaneously predict the prognosis of patients with BLCA and NSCLC.

### 3.7 Analysis of prognostic models constructed based on PRR11 co‐expressed genes in correlation with the immune microenvironment of BLCA and NSCLC

**FIGURE 7:**
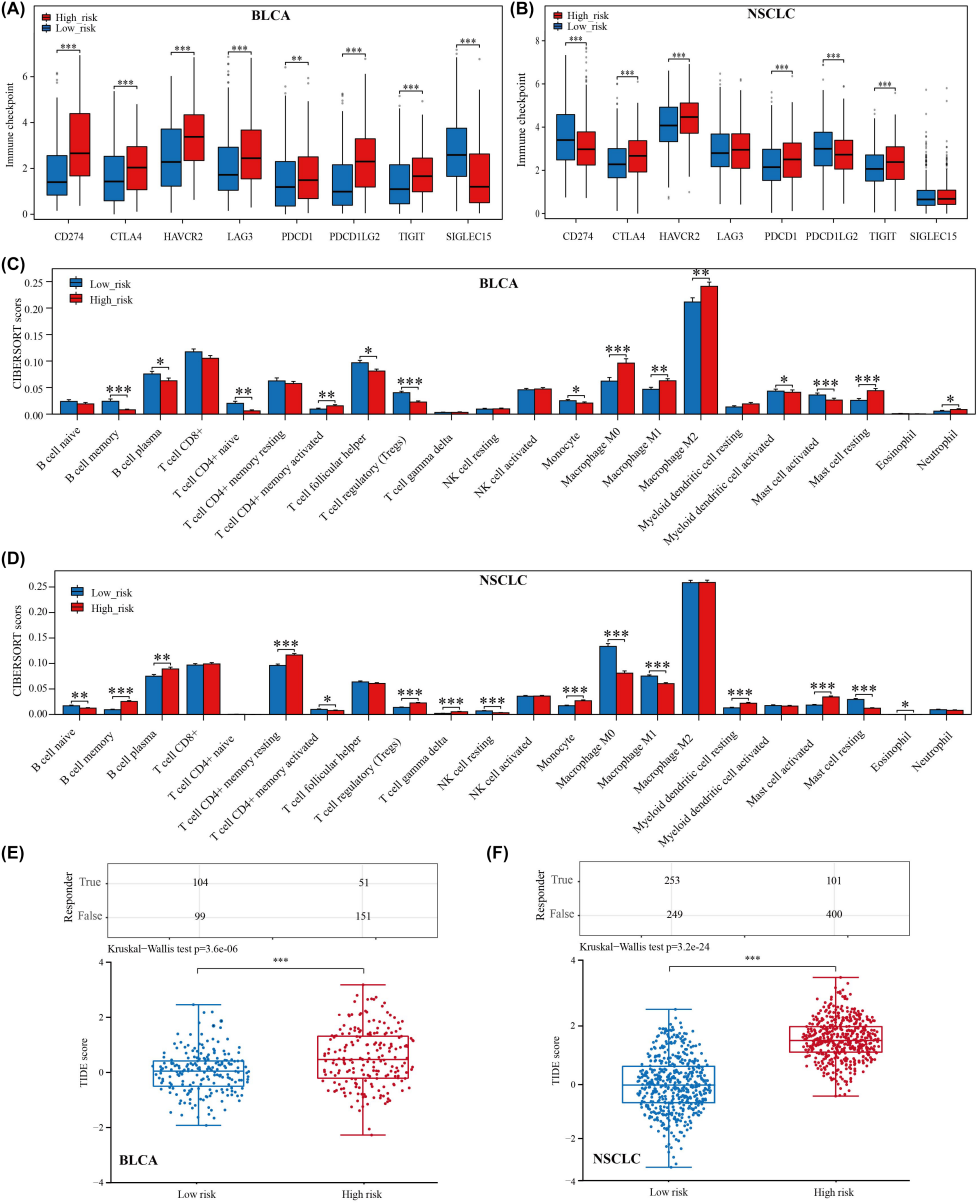
Analysis of prognostic models correlating with immunotherapy for BLCA and NSCLC. (A) Differential expression of immune checkpoint genes in BLCA between high‐ and low‐risk groups. (B) Differential expression of immune checkpoint genes in NSCLC between high‐ and low‐risk groups. (C) Differences in scores of immune‐infiltrating cells in BLCA between high‐ and low‐risk groups. (D) Differences in scores of immune‐infiltrating cells in NSCLC between high‐ and low‐risk groups. (E) Analysis of response to immune checkpoint inhibitor therapy in BLCA between high‐ and low‐risk groups using the TIDE algorithm. (F) Analysis of response to immune checkpoint inhibitor therapy in NSCLC between high‐ and low‐risk groups using the TIDE algorithm. *p < 0.05, **p < 0.01 and ***p < 0.001.

First, we analysed the differences in the expression of immune checkpoint‐related genes between the high‐ and low‐risk groups. The results revealed that all immune checkpoint genes were significantly different, except for SIGLEC15, which expression was not significantly different between the high‐ and low‐risk groups (Figure 7A,B). Subsequently, we analysed the correlation of immune‐infiltrating cells between the high‐ and low‐risk groups using the CIBERSORT algorithm. We found significant differences in memory B cells, plasma B cells T‐cell CD4+ memory activated, Treg, resting NK cells, monocytes, M0 macrophages, M1 macrophages, activated mast cells, and resting mast cells in both BLCA and NSCLC between the high‐ and low‐risk groups (Figure 7C,D). Finally, we analysed the responsiveness to immune checkpoint inhibitor treatment in the high‐risk and low‐risk groups; in both BLCA and NSCLC, patients in the high‐risk group had higher TIDE scores and immune checkpoint inhibitors were less effective in treating patients in the high‐risk group (Figure 7E,F). This might explain the poor prognosis of patients in the high‐risk group.

### 3.8 PRR11 regulates PDL1 through interaction with SPDL1

**FIGURE 8:**
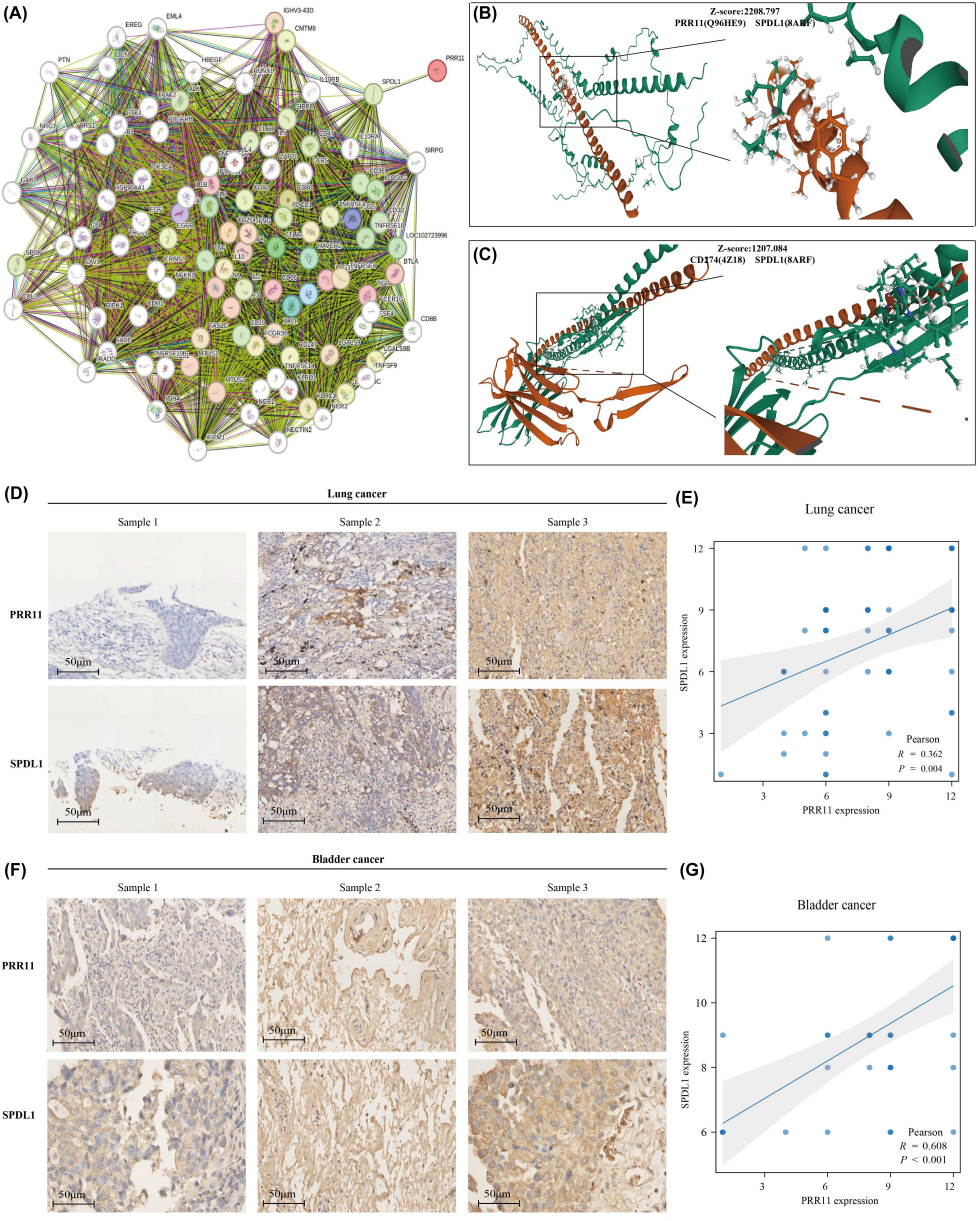
PRR11 and SPL1 are significantly correlated in both BLCA and NSCLC. (A) Interaction network of PRR11 and PDL1. (B) Molecular docking map of PRR11 and SPDL1. (C) Molecular docking map of PDL1 and SPDL1. (D) Expression of PRR11 and SPDL1 in lung cancer. (E) Correlation analysis of PRR11 and SPDL1 in lung cancer. (F) Expression of PRR11 and SPDL1 in bladder cancer. (G) Correlation analysis of PRR11 and SPDL1 in bladder cancer.

To investigate the potential mechanism underlying the regulation of PDL1 by PRR11, we used the STRING website to identify the interacting genes shared by PRR11 and PDL1. An interplay network diagram was constructed (Figure 8A). Our analysis revealed that SPDL1 is a reciprocal gene common to both PRR11 and PDL1, with an interaction score of 0.15. To further validate the relationship between PRR11, SPDL1 and PDL1, we performed molecular docking to examine their correlations at the structural level. Remarkably, we observed a strong correlation between PRR11 and SPDL1, as well as between PDL1 and SPDL1 (Figure 8B,C). Additionally, we analysed the correlation between SPDL1 and PRR11 expression in 60 lung and 41 bladder cancer tissues. The results consistently demonstrated a positive correlation between SPDL1 and PRR11 expression in both lung and bladder cancers (Figure 8D–G). In conclusion, our study provides evidence supporting the potential mechanism by which PRR11 regulates PDL1 expression in lung and bladder cancers. It is plausible that PRR11 influences the immunotherapy of these tumours through its interaction with SPDL1, thereby regulating the expression of PDL1.

### 3.9 The hsa‐miR‐200b‐3p/PRR11/SPDL1 signalling axis simultaneously regulates PDL1 expression in BLCA and NSCLC

**FIGURE 9:**
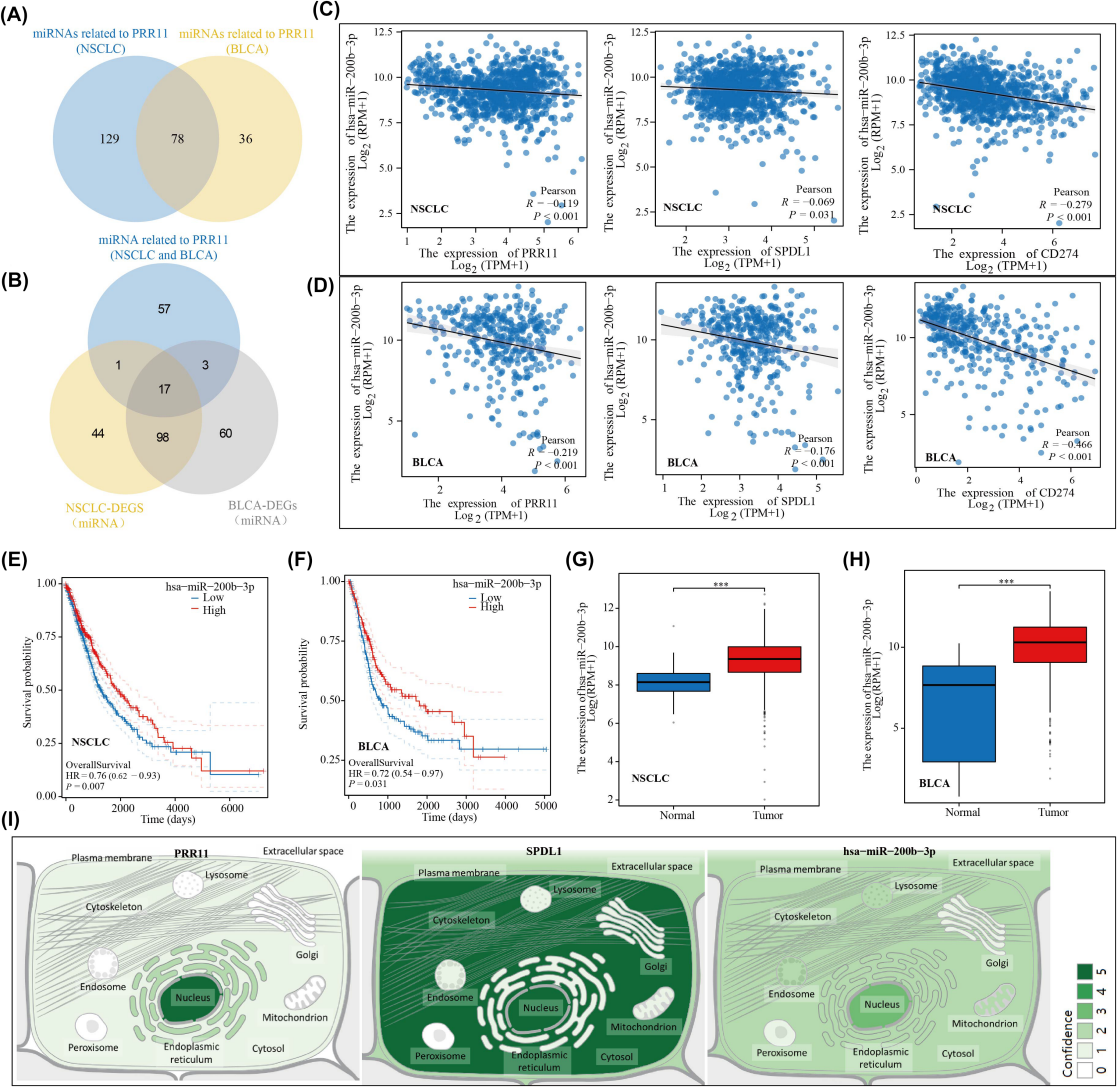
In NSCLC and BLCA, hsa‐miR‐200b‐3p is involved in the regulation of CD274 by PRR11. (A) PRR11‐related miRNAs in NSCLC and BLCA. (B) Differential miRNAs associated with PRR11 in NSCLC and BLCA. (C) hsa‐miR‐200b‐3p negatively correlates with PRR11, SPDL1, CD274 in NSCLC. (D) hsa‐miR‐200b‐3p negatively correlates with PRR11, SPDL1, CD274 in BLCA. (E, F) Prognostic KM curves of hsa‐miR‐200b‐3p in NSCLC and BLCA. (G, H) Differential expression of hsa‐miR‐200b‐3p in NSCLC and BLCA. (I) Cellular localisation of PRR11, SPDL1 and hsa‐miR‐200b‐3p. ***p < 0.001.

A total of 78 miRNAs were found to correlate with PRR11 in bladder and lung cancers. Among these 78 miRNAs, we identified 17 that exhibited differential expression in bladder and lung cancers compared with their corresponding normal tissues (Figure 9A,B). Interestingly, only hsa‐miR‐200b‐3p was negatively associated with PRR11, SPDL1 and CD274 expression in NSCLC and BLCA (Figure 9C,D). This suggests that hsa‐miR‐200b‐3p plays a role in the regulation of CD274 by both PRR11 and SPDL1 in bladder and lung cancers. Notably, low expression of hsa‐miR‐200b‐3p was significantly associated with poor prognosis in patients with NSCLC and BLCA (Figure 9E,F). Additionally, the expression of hsa‐miR‐200b‐3p was significantly higher in NSCLC and BLCA tissues than in the corresponding normal tissues (Figure 9G,H). Furthermore, analysis of cellular localization revealed that PRR11, SPDL1 and hsa‐miR‐200b‐3p were predominantly expressed in the nucleus (Figure 9I). In conclusion, our findings suggest that hsa‐miR‐200b‐3p acts upstream of PRR11 and is involved in the regulation of CD274 expression in NSCLC and BLCA.

## 4 DISCUSSION

In 2020, the GLOBOCAN study reported over 570,000 new cases of bladder cancer (BLCA), accounting for 2.1% of all cancer‐related deaths worldwide.^24^ Smoking is a significant risk factor for BLCA, as it is responsible for more than one‐third (36.8%) of all BLCA cases globally.^25^ Lung cancer is the leading cause of cancer‐related deaths globally, with approximately 1.6 million fatalities each year, whereas NSCLC accounts for approximately 85% of all lung cancer cases and its cure and survival rates remain low. Despite the well‐established correlation between smoking and lung cancer, 80.6%–81.9% of lung cancer deaths are attributed to cigarette smoke exposure.^26^ Immune system plays an important role in the development of cancers. Notably, the balance of cytokines significantly influences disease progression.^27^ Research has revealed that habitual smoking weakens the effectiveness of immune cells and molecules because of the presence of toxic substances, leading to the development of an immunosuppressive environment and tumour growth.^28^ Although extensive research has been conducted on the molecular mechanisms underlying smoking‐induced cancer, the evidence on how smoking promotes cancer development remains insufficient. Our research objective was to identify common immunotherapeutic targets for bladder and lung cancers in order to improve the prognosis of affected patients.

In this study, we identified 66 smoking‐associated differential genes that are common in NSCLC and BLCA. Gene enrichment analysis revealed that these genes primarily play a role in modulating cell cycle and cellular senescence. Previous studies have evidenced that smoking accelerates NSCLC progression by affecting cell cycle.^29^ Cigarette smoke damages DNA and impairs the mechanisms that control cell‐cycle checkpoints and DNA repair pathways.^30^ Additionally, it has been demonstrated that cigarette smoke extracts induce premature cellular senescence in individuals with chronic obstructive pulmonary disease by activating the p53/p21 signalling pathway.^31^ Moreover, cigarette smoke extracts can cause apoptosis and elicit innate immune responses.^32^ In recent years, the concept of precision medicine has led to the categorization of trial participants into subgroups. A well‐known example is the molecular categorization of breast cancer, which reveals distinct pathogenic mechanisms and clinical prognostic features in different subgroups.^33^ In our study, we classified patients with NSCLC and BLCA into three clusters to investigate the relationship between smoking status, prognosis and immunotherapy.^34^ The complex and dynamic crosstalk between tumour cells and TME has a crucial role in tumour growth, invasion and metastasis.^35^ We found a significant correlation between smoking‐related genes and immune‐infiltrating cells, as well as immune checkpoints, suggesting a close association between smoking‐related genes and the immune microenvironment in NSCLC and BLCA. Notably, PRR11 has emerged as the only smoking‐associated gene that can serve as a prognostic marker in both NSCLC and BLCA. The prognostic value and relevance of PRR11 in lung adenocarcinoma have been previously analysed.^36^ The growing research on TME has indicated that tumour‐infiltrating immune cells play a critical role in cancer progression and aggressiveness.^37^ Our study aimed to explore the immune infiltration relevance of PRR11 in lung cancer using the CIBERSORT algorithm in combination with single‐cell analysis. Our findings suggest that PRR11 plays a role in the immune infiltration of lung cancer by regulating monocytes and M0 macrophages. Additionally, we observed a significant association between PRR11 and PDL1, an immune checkpoint marker, in both NSCLC and BLCA. This suggests that PRR11 may potentially contribute to immunotherapy for NSCLC and BLCA by modulating PDL1 expression.

The development and immune evasion of cancer stem cells (CSCs) pose limitations to the effectiveness of current anticancer therapies.^38^ Stem cells release various cytokines, chemokines, growth factors and extracellular matrix (ECM) molecules that possess immunosuppressive and inflammatory modulatory properties. These molecules act via autocrine or paracrine pathways to exert immunomodulatory effects.^39^ In addition, the role of cigarettes in regulating tumour cell stemness has been demonstrated in kidney and lung cancers.^40^, ^41^ Through gene enrichment analysis, we discovered that PRR11 was associated with cancer stemness characteristics in both NSCLC and BLCA. In the NSCLC and BLCA samples, the PRR11 high‐expression group exhibited higher stemness scores, indicating a positive correlation between PRR11 and tumour stemness characteristics. To further investigate the role of PRR11 in the prognosis and immune microenvironment of NSCLC and BLCA, we constructed prognostic models based on PRR11 co‐expressed genes. The prognostic model developed for bladder cancer was also applicable to lung cancer, highlighting the consistent prognostic value of PRR11 in NSCLC and BLCA. PRR11 controls the expression of PDL1 in both NSCLC and BLCA. To investigate the underlying mechanisms, we used STRING to analyse genes that co‐interact with PRR11 and PDL1. Among these, we identified SPDL1 as a gene that co‐interacts with both PRR11 and PDL1. Interestingly, we observed a consistent positive correlation between PRR11 and SPDL1 expression in both the NSCLC and BLCA samples. To further validate this interaction, we used molecular docking to examine the molecular structure of the interaction between PRR11 and SPDL1. Additionally, we confirmed the correlation between PRR11, SPDL1 and PDL1 in NSCLC and BLCA tissue microarrays. To investigate the potential involvement of miRNAs upstream of PRR11 in the regulation of PDL1, we examined the association of hsa‐miR‐200b‐3p with PRR11, SPDL1, and PDL1 expression in NSCLC and BLCA. Our findings revealed a negative correlation between hsa‐miR‐200b‐3p and PRR11, SPDL1, and PDL1 in both NSCLC and BLCA. Furthermore, we identified hsa‐miR‐200b‐3p as a promising prognostic marker for NSCLC and BLCA. Overall, our study demonstrated consistent regulation of PDL1 by hsa‐miR‐200b‐3p/PRR11/SPDL1 in both NSCLC and BLCA.

## 5 CONCLUSION

In this study, we conducted a comprehensive analysis to investigate the prognostic and immunotherapeutic significance of smoking‐related genes in NSCLC and BLCA. Our findings reveal that PRR11, a commonly observed prognostic gene in both NSCLC and BLCA, plays a crucial role in regulating PDL1 through its interaction with SPDL1. Additionally, we discovered that hsa‐miR‐200b‐3p is involved in the regulation of PDL1 via the PRR11/SPDL1 pathway in NSCLC and BLCA.

## AUTHOR CONTRIBUTIONS

**YaXuan Wang:** Data curation (lead); formal analysis (lead); writing – original draft (lead). **HaiXia Zhu:** Investigation (equal); writing – original draft (equal). **Lu Zhang:** Formal analysis (equal); writing – original draft (equal). **JiaXing He:** Investigation (equal); validation (equal). **Ji Bo:** Data curation (equal); supervision (equal). **JianShe Wang:** Investigation (equal); validation (equal). **BeiChen Ding:** Validation (equal); writing – review and editing (equal). **MingHua Ren:** Funding acquisition (lead); resources (equal); writing – review and editing (equal).

## CONFLICT OF INTEREST STATEMENT

The authors declare that the research was conducted without any commercial or financial relationships that could be construed as a potential conflict of interest.

## Data Availability

The datasets were obtained from the TCGA database (https://portal.gdc.cancer.gov/) and TISCH (http://tisch.comp‐genomics.org/home/) database.
